# Prevalence and risk factors of antibodies towards HLA Class I and Class II in Malaysian renal transplant candidates

**DOI:** 10.1186/s12882-023-03085-6

**Published:** 2023-02-24

**Authors:** Norfarhana Khairul-Fahmy, Jamiila Ismail, Bee Tee Koay, Muhammad Zhafri Md-Zakariah, Salawati Mansor, Nordalila Zulkifli, Siti Fatimah Mat-Ali, Rozinah Mohamed, Norhazlin Mustafa, Masita Arip

**Affiliations:** 1 Transplantation Immunology Unit, Allergy and Immunology Research Centre, Institute for Medical Research, National Institutes of Health, Ministry of Health Malaysia, Shah Alam, Selangor 40170 Malaysia; 2 Allergy and Immunology Research Centre, Institute for Medical Research, National Institutes of Health, Ministry of Health Malaysia, Shah Alam, Selangor 40170 Malaysia

**Keywords:** Antibody-mediated rejection (AMR), HLA antibody, Renal allograft, Panel reactive antibody (PRA), Prevalence, Transplantation

## Abstract

Antibody-mediated rejection (AMR) still persists as the major hurdle towards successful renal allograft survival. This paper aims to report on the HLA antibody landscape of renal transplant candidates in Malaysia. A total of 2,219 adult samples from 2016 to 2019 were analysed for anti-HLA antibodies using solid-phase assay. Our findings highlight the prevalence and risk factors for antibodies against HLA antigens in renal transplant settings, which could be beneficial for selecting compatible recipients from deceased organ donors. To the best of our knowledge, this study is the first to demonstrate that ethnic Malay and Chinese showed significantly higher prevalence of anti-HLA antibodies. Based on our multivariate analysis: (i) female gender was associated with higher risk for panel reactive antibodies (PRAs) against Class I, Class II, and Class I and II (*p* < 0.001); (ii) older patients (≥ 38 years old) were associated with higher risk of positivity against Class I, Class II and Class I and II (*p* < 0.001); (iii) Malays showed significant association with Class II antibodies (*p* = 0.035); Chinese patients presented with higher risk of PRA positivity against Class II (*p* < 0.001) and Class I and II (*p* = 0.01); Indians were significantly associated with higher risk of HLA antibody sensitization against Class I (*p* = 0.022), Class II (*p* = 0.026) and Class I and II (*p* = 0.05). Thus, our findings suggested that female gender, older age (≥ 38 years old) and ethnicity may serve as independent risk factors for HLA antibody sensitization in adult renal transplant candidates.

## Introduction

Renal transplantation has been hailed as the ultimate treatment for any irreversible renal disease or renal failure. In comparison to dialysis, transplantation can improve life expectancy, provide better quality of life and reduce the cost for health care system [1]. Nevertheless, despite having better patient selection and management since the last decade, antibody-mediated rejection (AMR) still persists as the leading cause of long-term renal allograft failure [2, 3]. Alloantibodies against human leukocyte antigens (HLA), mainly carried out by donor specific antibodies (DSAs), are the main determinant for such rejection events, which can be present as pre-formed antibodies before the time of transplant or as *de novo* antibodies post-transplant. The presence of alloantibodies is regularly attributed to pregnancy, blood transfusion and history of transplantation. Patients with such characteristics are commonly termed as sensitized patients. This group of patients tend to have extended waiting times while listed for solid-organ transplantation [4] and the more sensitized they are, the less the chances of getting a compatible transplant in shorter period of time [5].

Recent years have seen huge leaps in the advancements of sensitive methods for alloantibody detection in clinical transplantation. The solid phase assay utilizing polystyrene or magnetic beads coated with HLA molecules, is a widely used method nowadays to identify the presence of DSAs. Quantification of DSAs is measured by mean fluorescence intensity (MFI) values. Different transplant centres utilize varying cut-off values to determine DSA positivity due to naturally occurring interferences on solid phase assay, but studies have suggested that a positive cut-off of 1,000 to 1,500 MFI would indicate a higher risk of rejection [6]. However, accurate interpretation of HLA antibody test analysis also relies much on patients’ clinical history, cross-reactive groups (CREGs) and HLA antigen distribution and coverage within a certain population [7].

In Malaysia, the numbers of incident and prevalent dialysis patients continue to increase significantly over the last 10 years, with 167 and 706 per million population (pmp) in 2008 to 216 and 1,363 pmp in 2018, respectively [8]. From these patients, approximately 90% were on hemodialysis and the rest were on peritoneal dialysis [8]. As with many other countries, diabetes mellitus remained the number one cause for end-stage renal disease (ESRD) in new dialysis patients and Malaysia ranks second in the world to have incident cases of treated ESRD attributed to diabetes [9]. Despite the continuous increase in dialysis population, the number of new kidney transplantation rate had remained very low throughout the years. Malaysia’s transplant prevalence rate was at 3 pmp in 2018 and local cadaveric donor transplantation contributed only 12% of the transplants performed that same year [8]. Although most kidney transplantations performed were from living related donors, this unfortunately still does not resolve the issue of organ shortage and the increasing number of dialysis patients.

Due to this situation, selection of the most compatible patient is crucial in determining the best outcome for both patient and graft survival. Patients that are pre-sensitized with HLA antibodies pose a drastic rise in the risk of hyper-acute rejection and allograft loss. Despite the significant advances in immunosuppressive therapy and better knowledge on the development of HLA antibodies, transplantation in sensitized patients remains challenging. To date, data on the prevalence of HLA antibodies among the Malaysian transplant patients are still scarce. Hence, this paper aims to report on the HLA antibody landscape of transplant candidates that were analyzed retrospectively from year 2016 till 2019 in Malaysia.

## Methods

A total of 2,219 adult samples were sent and tested for HLA antibody test at Transplantation Immunology Unit, Allergy & Immunology Research Centre, Institute for Medical Research (IMR), Malaysia from year 2016 till 2019. The laboratory is ISO 15189:2014 certified and routinely participates in both internal and external quality assurance programmes.

All samples were initially screened using a commercially available Luminex LABScreen™ Mixed Assay (One Lambda, Canoga Park, CA, USA) that consists of colour-coded microbeads coated with purified HLA Class I, Class II and MHC Class I chain-related A (MICA) antigens. Positive samples for any HLA antibody classes on the mixed assay will be tested with LABScreen™ PRA Class I or LABScreen™ PRA Class II, respectively and/or the LABScreen™ Single Antigen HLA Class I or LABScreen™ Single Antigen HLA Class II (One Lambda, Canoga Park, CA, USA). The PRA kits determine the percentage of PRA and identify antibody profiles using a panel of Class I or Class II HLA antigens [10] depending on the kits used. Meanwhile, single antigen-bead based assay utilizes recombinant specific type of HLA antigens coated onto each microbead that allows a precise determination of antibodies against HLA antigens, with which the LABScreen™ Single Antigen is more effective in overcoming antibodies reactive to one or more dominant epitopes that can mask the presence or additional antibody specificities [11].

In principle, serum samples were incubated with microbeads from respective kits and any unbound HLA antibodies will then be washed away. A conjugate antibody, R-phycoerythrin conjugated anti-IgG was then added, where any unbound conjugate will be washed away before acquisition of bound HLA antibodies using LABScan3D™ (Luminex® FLEXMAP 3D®) (One Lambda, Canoga Park, CA, USA). Output data from LABScan3D™ was further analysed using HLA Fusion™ software (One Lambda, Canoga Park, CA, USA), where the individual beads were analyzed and results were reported semi-quantitatively as MFI.

Considerations for determining positivity includes: (i) negative control values – less than 500 MFI for each run; (ii) positive control values – more than 3,000 MFI; (iii) positive-to-negative control ratio values of more than 2.0; (iv) MFI for each HLA type; and (v) CREGs of HLA antigen. For each HLA type, the calculated MFI values were normalized against the internal negative control beads and the negative serum controls. For samples that showed comparable MFI levels across several HLA types, CREGs were applied to determine positivity towards specific or multiple public epitopes. As a general rule, MFI of approximately ≥ 1,000 is considered as positive after taking into consideration of the above factors. The cut-off of 1,000 MFI was determined through our laboratory validation study, which included both the clinical samples and the external quality assurance samples by UK NEQAS for Histocompatibility and Immunogenetics. For statistical analysis, continuous data were described as mean ± standard deviation (SD) (range) and categorical data were presented as percentage (%). Comparison between positive and negative PRA groups were tested by Pearson chi-square tests and Fisher’s exact tests where appropriate. Binary logistic regression tests with univariate and multivariate analyses were performed to determine the relationship for a single significant parameter and multiple significant parameters, respectively. A two-sided *p* value of less than 0.05 was considered statistically significant. Statistical calculations were performed with IBM SPSS Statistics for Windows, version 26.0 [12].

## Results

### Patients’ characteristics

In this study, 2,219 adult samples were collected from 2016 to 2019 and compiled for analysis of anti-HLA antibodies. Table 1 outlines the demographics of these patients. The mean age of patients was 37.95 ± 10.93 (range, 18–73). As Malaysia is a multi-ethnic country, the demography of each major ethnic group was also taken into consideration – 60.0% were of Malay ethnicity, 22.9% were Chinese, 9.6% were Indians and the remaining 7.5% were from other minor ethnicities that include the Iban, Dusun, Kadazan, Bidayuh and a few others.

**Table 1:** Demographic characteristics

Characteristics	
All patients	2,219
Age (range), years	37.95 ± 10.93 (18–73)
Gender, n (%)	
Male	1,164 (52.5%)
Female	1,055 (47.5%)
Ethnicities, n (%)	
Malay	1,332 (60.0%)
Chinese	507 (22.9%)
Indian	213 (9.6%)
Others	167 (7.5%)
PRA	
Class I+/Class II-, n (%)	710 (33.0%)
Class I-/Class II+, n (%)	595 (27.7%)
Class I+/Class II+, n (%)	363 (16.9%)

Age is expressed as mean ± SD. Abbreviation: PRA, panel reactive antibody; +, positive; -, negative

### Prevalence of panel reactive antibodies (PRAs) for Class I and/or Class II

Among these patients, only 97% patients’ test results can be analyzed – the remaining 3% of samples failed due to various reasons such as high background, failed negative or positive control and/or poor sample quality. As shown in Table 1, 710 (33.0%) patients were positive for Class I and 595 (27.7%) were positive for Class II. Three hundred and sixty-three (16.9%) patients showed positivity for both Class I and Class II.

### Risk factors associated with PRAs for Class I and/or Class II

Factors including gender, age and ethnicities that may influence the positive rates of PRAs against HLA Class I and Class II, were examined using univariate analysis (Table 2). Gender, age and ethnicities were found to be significantly associated with antibodies against Class I+/Class II- and Class I+/Class II+. However, for Class I-/Class II + antibodies, only gender and age were significantly considered as risk factors. Multivariate analysis showed the following: (i) female gender was significantly associated with higher risk of PRA sensitization against all three categories; (ii) older patients (≥ 38 years old) were associated with higher PRA risk towards all categories; (iii) Malay patients were significantly more likely to have PRA sensitization towards Class I-/Class II + only, Chinese showed significant higher risk towards Class I-/Class II + and Class I+/Class II + and Indians were significantly associated with higher risk against all three categories (Table 3).

**Table 2:** Univariate analysis for PRA in 2,150 adult samples for PRA testing

		Class I+/Class II-		Class I-/Class II+		Class I+/Class II+	
		*n* (%)	*p*-value	*n* (%)	*p*-value	*n* (%)	*p*-value
Gender							
	Male (n = 1120)	290 (25.9%)	< 0.001	267 (23.8%)	< 0.001	146 (13.0%)	< 0.001
	Female (n = 1030)	420 (40.8%)		328 (31.8%)		217 (21.1%)	
**Age**							
	< 38 years (n = 1117)	298 (26.7%)	< 0.001	272 (24.4%)	< 0.001	144 (12.9%)	< 0.001
	≥ 38 years (n = 1033)	412 (39.9%)		323 (31.3%)		219 (21.2%)	
**Ethnicity**							
	Malay (n = 1295)	379 (29.3%)	< 0.001	351 (27.1%)	ns	196 (15.1%)	0.003
	Chinese (n = 493)	187 (37.9%)		151 (30.6%)		107 (21.7%)	
	Indian (n = 206)	90 (43.7%)		61 (29.6%)		40 (19.4%)	
	Others (n = 156)	54 (34.6%)		32 (20.5%)		20 (12.8%)	

Abbreviations: PRA, panel reactive antibody; +, positive; -, negative; ns, not significant

**Table 3:** Logistic regression analysis of PRA in 2,150 adult samples for PRA testing

		Class I+/Class II-			Class I-/Class II+			Class I+/Class II+		
		**Adjusted OR**	**95% CI**	***p*** **-value**	**Adjusted OR**	**95% CI**	***p*** **-value**	**Adjusted OR**	**95% CI**	***p*** **-value**
Gender (Female vs. Male)		2.041	1.694–2.461	< 0.001	1.524	1.258–1.846	< 0.001	1.839	1.459–2.324	< 0.001
Age (≥ 38 years vs. < 38 years)		1.753	1.455–2.114	< 0.001	1.387	1.144–1.682	< 0.001	1.730	1.371–2.189	< 0.001
Ethnicities										
	Malay	0.874	0.614–1.257	ns	1.555	1.044–2.378	0.035	1.347	0.837–2.277	ns
	Chinese	1.231	0.840–1.819	ns	1.782	1.163–2.795	< 0.001	1.983	1.199–3.424	0.010
	Indian	1.677	1.081–2.614	0.022	1.753	1.075-2.900	0.026	1.802	1.010–3.302	0.050
	Others		1			1			1	

Abbreviations: PRA, panel reactive antibody; OR, odds ratio; CI, confidence interval; +, positive; -, negative; ns, not significant

## Discussion

In this study, we demonstrated that females and older age were related to having antibodies against HLA antigens, where these results were in agreement with findings from previous studies [13–18]. Ethnicity also may play a role as an independent risk factor for HLA sensitization. To the best of our knowledge, this study is the first to demonstrate that major ethnics in Malaysia pose significant risk of having anti-HLA antibodies. Our findings highlight the prevalence and risk factors for antibodies against HLA antigens in renal transplant settings and could be beneficial especially in the selection of compatible recipients for deceased organ donors.

The association between anti-HLA antibodies and gender has previously been reported in many studies. For instance, Hung et al. highlighted that PRA-positive ESRD patients were mainly females (73.1% females vs. 26.9% males, p = 0.000) and these patients significantly showed to have histories of pregnancy and transfusion [13]. Similarly, Hyun et al. showed that females displayed a higher PRA-positive rate compared to males (60.3% vs. 34.2%, p < 0.001) [14], regardless of the presence or absence of other sensitization events. Most of the previous studies in overall, highlighted that females tend to have higher sensitization to HLA antibodies. This is highly expected due to the nature of females experiencing parity events in life. Most notably, the immunization frequency of HLA antibodies increases with the number of pregnancies and the number of children [15]. Thus, our results support previous findings suggesting that female gender can be regarded as an independent risk factor for the presence of antibodies against HLA antigens.

In the present study, we have demonstrated that older age is a risk factor for antibody sensitization towards HLA antigens. The cut-off of 38 years old was based on the mean age of our sample population (37.95 years ± 10.93). Many other studies nonetheless, showed slightly older mean age than the current study, which on average, were more than 40 years old – Betjes et al. reported a mean age of 43.8 years old in 21.8% DSA positive kidney recipients [16], Ilagan et al. showed a mean age of 45.6 years old in 25.6% PRA-positive patients [17], while Karahan et al. reported a mean age of 42.01 years old in 17.3% PRA-positive Turkish ESRD patients [18]. The difference between our study and others might be attributed to lower number of patients tested that were of 65 years and above; the majority enrolled in this study were in the range of 30–59 years of age. This is in parallel with Malaysia’s current clinical practice that disqualifies candidates above 60 years old for renal transplant therapy [19], thus testing less patients beyond that age for anti-HLA antibody sensitization. Immunologically, long-lived plasma cells (LLPCs) can produce HLA alloantibodies for a lifetime and this phenomenon plays a crucial role in antibody-mediated rejection [20]. Older patients usually have longer exposure to sensitization events, thus triggering the PRA positivity and subsequently can compromise the longevity of graft survival.

Limited studies have shown the association between ethnicity and HLA sensitization, especially of the Asian ancestry. Most of the available studies were more focused on the Caucasians and other populations in the Western countries. For instance, Lucas et al. found that mean frequencies of antibodies to HLA antigens in blacks on average were higher than, or comparable to those of whites for all HLA loci, although the results were not statistically significant [21]. Similarly, in a study of heart transplant recipients, Morris et al. also reported that blacks showed significantly higher peak PRA and were more likely to be sensitized than all other groups (whites, Hispanics and Asians) [4]. Among the Asians itself, we could not find any studies that compare the association of anti-HLA antibodies between ethnicities. In the current study, we found significant associations of HLA sensitization between ethnicities through the univariate logistic regression analysis (Table 2). Our multivariate logistic regression (Table 3) revealed that Malays only showed significance against sensitization towards Class II, whereas Chinese significantly showed association with Class II and Class I and II together. Indians were significantly associated with antibody sensitization across all categories. The significant association among the Indians in our population were coherent with the findings from Chauhan et al. indicating that Indians from their centre also showed high prevalence of both Class I and Class II HLA antibodies [22]. Nevertheless, the associations with the Malays and Chinese have not been reported before. We believe that this is the first study to demonstrate that both ethnics independently displayed significant association with the presence of anti-HLA antibodies.

The biological mechanism in this matter remains unclear. However, we speculate that these associations are related to the higher susceptibility of the Asians towards systemic lupus erythematosus (SLE) [23]. The prevalence of SLE in Asians generally falls within 30–50 per 100,00 individuals [24]. In Malaysia, ethnic Chinese had the highest prevalence rate of 57 per 100,000 individuals, followed by the Malays with 33 per 100,000, and the Indians with 14 per 100,000 [25]. Several studies have highlighted significant associations of HLA genes in disease pathogenesis within the Chinese and Malays [26, 27]. This is also concurrent with the fact that renal involvement was high among the SLE patients in Malaysia [25]. Thus, it is possible that our cohort in this study were presented with SLE and glomerulonephritis as their primary renal disease, although these diseases only contributed to less than 3.5% of the new dialysis patients in Malaysia [8]. Further studies are highly needed to prove such association.

There are several limitations to the present study. First, it was conducted in a single-centre setting. Being the national referral centre for HLA testing that caters diagnostic services to all transplantation hospitals in Malaysia, it is deemed unavoidable with the current local setting. Secondly, we did not investigate the history of sensitization events in all these patients, nor did we look into the antibodies against any specific loci, in which this information would give a deeper insight into the risk factors. Most of our patients have multiple episodes of blood transfusions performed at various centres, where in some centres, these events were not incorporated into the national dialysis registry, hence making it difficult to keep track of sensitizing events in each patient. Therefore, a more detailed analysis of the patients’ clinical background, especially within the Malaysian population, is warranted. Additionally, ethnicity is by self-report. It is possible that patients with mixed ethnicities may have confounded the findings.

In summary, the present study suggested that female gender, older age (≥ 38 years old) and ethnicity are independent risk factors for HLA antibody sensitization in adult renal transplant candidates. These findings could help in assisting for the selection of a suitable donor, especially for those patients on the waiting list as well as supporting clinical monitoring of HLA antibodies pre- and post-transplantation.

## Data Availability

The datasets generated during and/or analysed during the current study are available from the corresponding author on reasonable request.
